# Clinical experience in the management of patients with Graves' orbitopathy treated according to the novel protocol of glucocorticoid therapy designed in the Department of Endocrinology, Metabolism and Internal Medicine of Poznan University of Medical Sciences

**DOI:** 10.1186/1756-6614-6-S2-A54

**Published:** 2013-04-05

**Authors:** Jerzy Sowiński, Nadia Sawicka, Agnieszka Skiba, Marek Ruchała

**Affiliations:** 1 Department of Endocrinology, Metabolism and Internal Medicine, Poznan University of Medical Sciences, Poznan, Poland

## Introduction

Graves' orbitopathy, despite of the results of recent researches concerning pathomechanism of the disease, is still a therapeutic challenge. Corticosteroids are the treatment of choice for active, moderate to severe Graves' orbitopathy.

The aim of the study is to evaluate the effects of treatment of patients with GO with novel protocol of steroidotherapy designed and introduced in Department of Endocrinology, Metabolism and Internal Medicine. The management protocol consists of 3 g of methylprednisolone administered intravenously followed with methylprednisolone injected intramuscularly in divided doses every 3 weeks (total dose of methylprednisolone is 3.6 g).

## Material and methods

The study group consisted of 50 patients. Assessment of efficacy of therapy was performed before and immediately after the therapy and 6 months later. Thyroid function parameters (TSH, FT4, FT3), titers of thyroid autoantibodies (TRAb, TPOAb, TgAb), thyroid volume (V) were analyzed. Moreover, ophthalmological findings (soft tissue involvement, proptosis, diplopia, clinical activity score, visual acuity) and disease activity on magnetic resonance imaging of orbits were evaluated.

## Results

The therapy significantly improved the degree of soft tissue involvement, CAS, diplopia and decreased the autoimmune disease activity in oculomotor muscles. Moreover, significant decrease of TRAb titer was observed.

## Conclusions

To conclude, novel protocol of glucocorticoid therapy is effective and safe.

